# Photometric analysis of esthetically pleasant and unpleasant facial profile

**DOI:** 10.1590/2176-9451.19.2.066-075.oar

**Published:** 2014

**Authors:** Helena Nunes da Rocha Fortes, Thamirys Correia Guimarães, Ivana Mara Lira Belo, Edgard Norões Rodrigues da Matta

**Affiliations:** 1 Degree in Dentistry, Federal University of Alagoas (UFAL); 2 Specialist in Orthodontics, School of Dentistry - University of São Paulo/Bauru; 3 Adjunct professor, Federal University of Alagoas (UFAL)

**Keywords:** Esthetics, Photography, Orthodontics

## Abstract

**Objective:**

To identify which linear, angular and proportionality measures could influence a profile to be considered esthetically pleasant or unpleasant, and to assess sexual dimorphism.

**Methods:**

150 standardized facial profile photographs of dental students of both sexes were obtained and printed on photographic paper. Ten plastic surgeons, ten orthodontists and ten layperson answered a questionnaire characterizing each profile as pleasant, acceptable or unpleasant. With the use of a score system, the 15 most pleasant and unpleasant profiles of each sex were selected. The photographs were scanned into AutoCAD computer software. Linear, angular and proportion measurements were obtained using the software tools. The average values between groups were compared by the Student's t-test and the Mann-Whitney test at 5%.

**Results:**

The linear measures LL-S, LL-H, LL-E, LL-B and Pn-H showed statistically significant differences (p < 0.05). Statistical differences were also found in the angular measures G'.Pn.Pg', G'.Sn.Pg' and Sn.Me'.C and in the proportions G'-Sn:Sn-Me' and Sn-Gn':Gn'-C (p < 0.05). Differences between sexes were found for the linear measure Ala-Pn, angles G'-Pg'.N-Pn, Sn.Me'.C, and proportions Gn'-Sn:Sn-Me' and Ala-Pn:N'-Sn. (p < 0.05).

**Conclusion:**

The anteroposterior position of the lower lip, the amount of nose that influences the profile, facial convexity, total vertical proportion and lip-chin proportion appear to influence pleasantness of facial profile. Sexual dimorphism was identified in nasal length, nasofacial and lower third of the face angles, total vertical and nasal height/length proportions.

## Abstract (pt)

**Objetivo:**

identificar quais medidas lineares, angulares e de proporcionalidade influenciam para um perfil ser considerado agradável ou desagradável, e avaliar o dimorfismo sexual.

**Métodos:**

foram obtidas e impressas em papel fotográfico 150 fotografias padronizadas do perfil facial de acadêmicos de Odontologia de ambos os sexos. Dez cirurgiões-plásticos, dez ortodontistas e dez leigos responderam um questionário caracterizando cada perfil em agradável, aceitável ou desagradável. Com a utilização de um sistema de escore, foram selecionadas as fotografias dos 15 perfis mais agradáveis e dos 15 mais desagradáveis de cada sexo. As fotografias foram digitalizadas para o programa de computador AutoCAD, e medidas lineares, angulares e de proporcionalidade foram mensuradas utilizando-se ferramentas do programa. As médias obtidas entre os grupos foram comparadas pelos testes *t* de Student e de Mann-Whitney a 5%.

**Resultados:**

as medidas lineares Li-S, Li-H, Li-E, Li-B e Pn-H apresentaram diferenças estatisticamente significativas (p < 0,05). Os ângulos G'.Pn.Pg', G'.Sn.Pg' e Sn.Me'.C e as medidas de proporcionalidade G'-Sn:Sn-Me' e Sn-Gn':Gn'-C também apresentaram diferenças significativas (p < 0,05). Diferenças significativas entre os sexos foram identificadas na medida linear Ala-Pn, nos ângulos G'-Pg'.N'-Pn, Sn.Me'.C e proporções Gn'-Sn:Sn-Me' e Ala-Pn:N'-Sn (p < 0,05).

**Conclusão:**

o posicionamento anteroposterior do lábio inferior, a quantidade do nariz que influencia o perfil, a convexidade facial, a proporção vertical total e lábio-queixo parecem influenciar a agradabilidade do perfil facial. Dimorfismo sexual foi identificado na medida linear comprimento nasal, nos ângulos nasofacial e terço inferior da face e nas proporções vertical total e comprimento/altura nasal.

## INTRODUCTION

Facial esthetics is considered a significant factor with regard to the perceptions of society and individuals in relation to themselves. Additionally, it plays an important role in the assessment of personality and social acceptance.^1^ The ability in recognizing a beautiful face is innate,^2^ and the development of esthetic perception happens since childhood.^1,3^

A good facial esthetics is one of the factors that influences the judgement of beauty which is related to the individual's relationship with society, thus enhancing self-esteem.^1^ 80% of adults seeking orthodontic treatment for themselves or their children do so based on esthetic motivations, regardless of functional and structural conditions.^4^ Therefore, as the goal of most patients is having an esthetically pleasing face, orthodontic treatment plays an important role by modifying the position of teeth, bones and associated soft tissues.

In this context, orthodontic treatment should aim not only at correcting the position of teeth in the bone bases, but also at achieving the best possible facial configuration, associating a harmonious face with an ideal occlusion.^5^ It is known that the results obtained from the cephalometric normative values oftentimes do not correspond with the individual patterns of each patient.^1,6^ Many authors have attempted to define the facial features responsible for a pleasant facial esthetics and observed that the constituent parts of a facial profile, when in harmony and balance, were associated with facial pleasantness.^7^

Studying facial profile through soft tissues has become object of interest among professionals in the orthodontic field over the years. This analysis can be used to assess the face not only in terms of changes induced by orthodontic treatment or growth, but also facial esthetics,^8^ considered as the best determinant of orthodontic treatment outcomes.^9^

There are several methods used to analyze craniofacial morphology in facial profile, one of which is achieved at the expense of standardized photographs which occupy a prominent place in facial analysis, being routinely performed by most orthodontists.^5^ Photographs provide a good assessment of harmony between the external craniofacial structures, including the contribution of soft tissues, in addition to providing reliable measurements,^10^ allowing a quick capture of facial image, having long-term durability and the possibility of taking repeated measurements.^11^ Through photometric analysis of the facial profile, proportionality, angular and linear measurements can be obtained. To many researchers, these measures serve as a parameter to better define normal conditions, harmony and balance of the profile.^12^

Several studies have tried to find the characteristics responsible for facial pleasantness, classifying facial profiles by subjective analysis of photographs, noting orthodontists and laypeople's point of view.^1,3,13,14^ Others have sought to study some characteristics that can influence the esthetics of the profile,^9,12,15^ however, what makes a facial profile to be considered more esthetic is not yet fully understood by all clinicians.^16^

Therefore, this study aimed at assessing through standardized photographs which linear, angular and proportionality measures differ from profiles classified as esthetically pleasant and unpleasant.

## MATERIAL AND METHODS

This research was approved by the Federal University of Alagoas Institutional Review Board and was registered under the number 007455/2009-43. The participants who were photographed as well as the evaluators signed a consent form.

For this study, 150 (75 males and 75 females) dental students, aged between 17 years and 9 months to 32 years old, were selected. In selecting the sample, the following inclusion criteria were applied: the subjects should be caucasian; mesofacial; should not wear orthodontic appliances or any other intraoral device that could influence the profile; and should not present facial asymmetry or evident vertical or sagittal discrepancy. Sample size was defined on the basis of convenience, gathering the largest number of students who agreed to participate in the study. Due to the difficulties of scientifically defining race and ethnicity, and because of the great racial mixture of Brazilian people, race was classified by skin color in Caucasian, Mongoloid and Melanoderm. This classification method is widely used in Orthodontics, and for this study, only Caucasians were selected.

**Figure 1:**
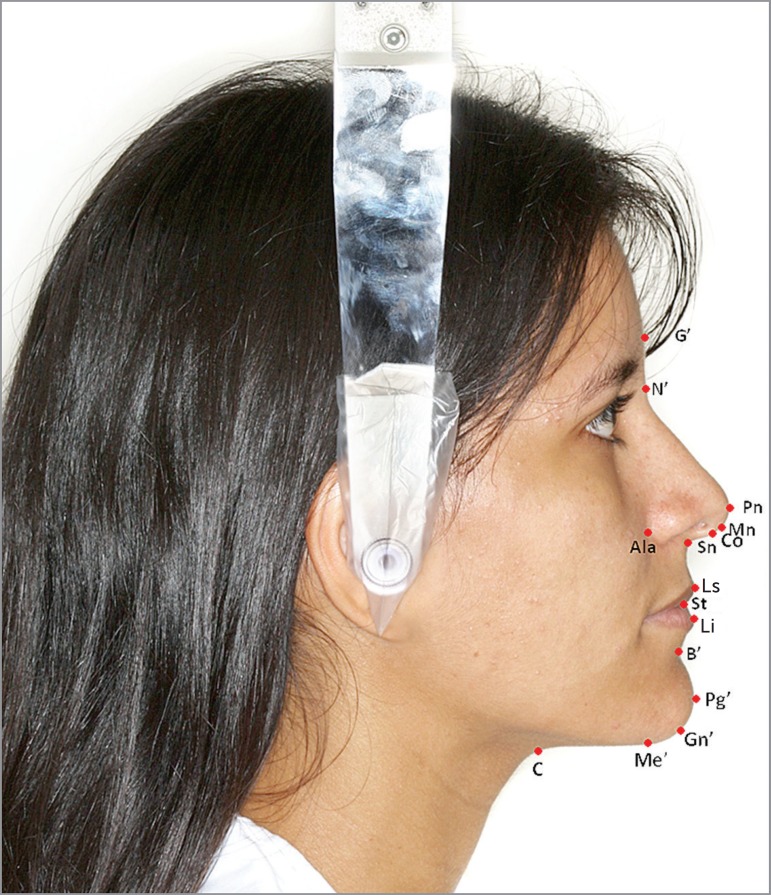
Photographic reference points.

Standardized photographs of the right profile were taken with a digital camera, (Canon Rebel, Tokyo, Japan), model Rebel EOS XS, 100 macro lens and macro ring lite flash model MR-14EX, (Canon, Japan). In order to standardize the photographs, they were obtained by a single operator, in the same environment, at the same distance between the research subject and the camera. Moreover, all the other photographic parameters were also standardized, namely: aperture f11, shutter speed 1/125 and ISO 200. Patients were at rest position, completely relaxed and positioned in a cephalostat (Fig 1).

The photographs were printed on Kodak glossy photo paper (Kodak do Brasil, São José dos Campos, Brazil), size 15x21, using a Noritsu 1501 professional printer (Noritsu Koki Co Ltd, Tokyo, Japan). They were divided into two books, one comprising male photographs and another comprising female photographs, which were delivered to the evaluators - 10 orthodontists, 10 plastic surgeons and 10 laypeople - at their workplaces. The evaluators had to answer to a questionnaire, and for each photograph there were three choices: esthetically pleasant, acceptable or unpleasant facial profile. After the questionnaires were collected, a score system was applied. The photographs classified as presenting an esthetically pleasant profile received a score of +1, acceptable profile was scored with 0 and unpleasant profile with -1. Thus, each photograph could receive a total score that ranged from -30 to +30. This scoring system was only used to select the 15 most pleasant and unpleasant photographs, with no descriptive statistics being performed on the data obtained, only the total sum of points was performed. This study did not assess the differences in perception of different groups of evaluators. The latter were used to ensure that the different concepts of facial esthetics of professional and lay groups were present in the selection of pleasant and unpleasant profiles. Afterwards, the 15 males and 15 females' photographs with the highest scores were selected as representative of an esthetically pleasant profile, whereas the 15 males and 15 females' with the lowest scores were selected as esthetically unpleasant. In selecting the esthetically pleasant facial profiles, should there be a draw score , the following tie-breakers were used in the following priority order: photograph with the highest number of +1 scores, with the greatest number of 0 scores and the lowest number of -1 scores; classification or not in the assessment of the 30 evaluators . In selecting the esthetically unpleasant facial profiles, should there be a draw score, the following tie-breakers were used in the following order of priority: photograph with the highest number of -1 scores, with the highest number of 0 scores and the lowest number of +1 scores; classification or not in the assessment of the 30 evaluators.

After that, the photographs were scanned on a HP G2410 scanner (Hewlett-Packard Company, Palo Alto, Cali, USA), under 300 dpi resolution, and saved in the AutoCAD 2007 software (Autodesk, Inc, San Rafael, Cali, USA).

To eliminate distortion between the actual size of the face and the size of the photograph, the metal screw of the cephalostat, which is well defined on the photograph, served as a reference. Its actual size was measured with the use of a digital caliper. The actual diameter of the screw of the cephalostat is 15.79 mm. Thus, a circle with the same diameter of the cephalostat screw was designed in the AutoCad software. Then, the images of the screws in each photograph were adjusted to fit the circle drawn in the software. Consequently, the measurements obtained in the software are equivalent to the actual measurements, thus, eliminating the need for obtaining a correction factor.

The facial points markings and measurements were performed by the same operator. The markings were done in two days and the measurements were taken within 6 days (10 photos a day) in order to avoid fatigue and, as a consequence, operator's error. The photographic reference points were selected according to previous studies conducted by Trevisan and Gil^8^ as well as Sutter and Turley,^17^ as shown in Figure 1.

**Figure 2:**
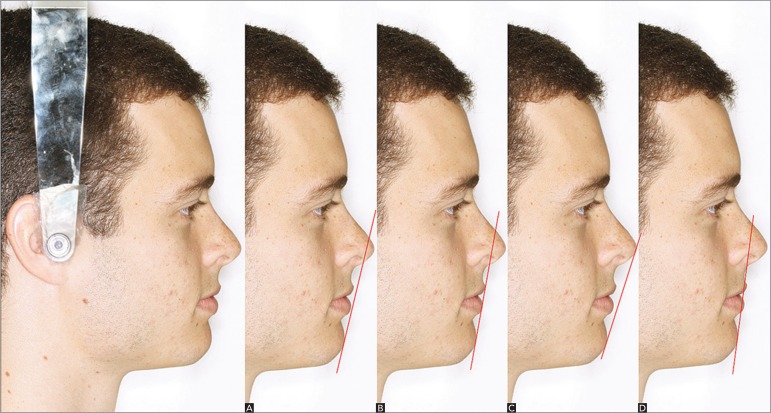
Reference lines - **A)** Steiner S line; **B)** Holdaway H line; **C)** Ricketts E line **D)** Burstone B line.

**Figure 3:**
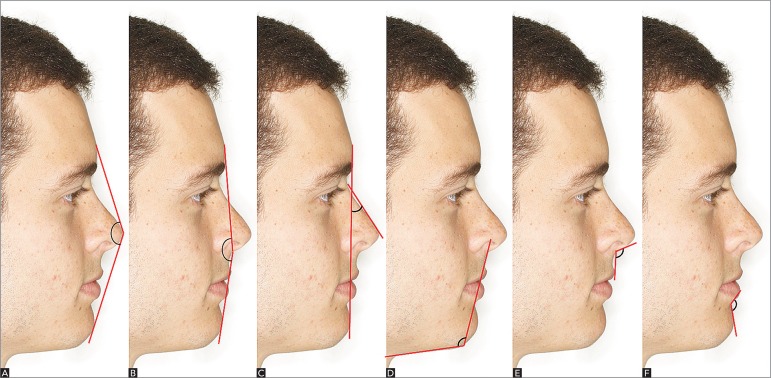
Angular measurements and reference lines: **A)** Total facial convexity angle (G'.Pn.Pg'); **B)** Facial convexity angle (G'.Sn.Pg'); **C)** Nasofacial angle (G'-Pg'.N'-Pn); **D)** Lower third angle (Sn.Me'.C); **E)** Nasolabial angle (UL.Sn.Co); **F)** Mentolabial angle (Pg'.B'.LL).

**Figure 4:**
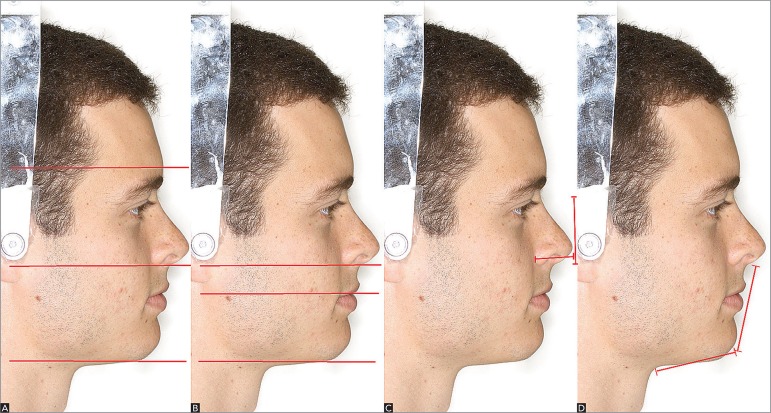
Measures of proportionality used in this study: **A)** Total vertical proportion (G'-Sn:Sn-Me'); **B)** Inferior third of the face proportion (Sn-St:St-Me'); **C)** Nasal height /length proportion (Ala-Pn:N'-Sn); **D)** Lip-chin proportion (Sn-Gn':Gn'-C).

For profile analysis, the reference lines used in the cephalometric analysis of Steiner, Holdaway, Ricketts and Burstone were drawn to obtain linear measurements that assess the distance from points UL, LL, and Pn to these lines, as shown in Figure 2. As for measurement of nasal length, the distance between the Ala-Pn points was measured. Angular and proportionality measurements were obtained according to Trevisan and Gil^8^ as well as Sutter and Turley^17^ using an AutoCAD software tool. Such measurements are shown in Figures 3 and 4, respectively.

### Statistical analysis

The data obtained were tabulated and statistically analyzed. Initially, the Kolmogorov-Smirnov normality test with a significance level set at 5% was used to evaluate the distribution pattern of the data. To compare the means of the variables with normal distribution, a parametric test (Student's t-test) was used, and for variables not normally distributed the non-parametric Mann-Whitney test was used, both with a significance level set at 5%.

### Method error

For intra-examiner error, 30 days after the measurements had been taken, 1/3 of the measures of all variables were repeated and the first and second measures were compared. The random error was calculated in accordance with the formula proposed by Dahlberg and advocated by Houston.^18^ The deviation values were acceptable below 1 mm for the linear measurements,and 1.5^o^ for angular measurements. The systematic error was analyzed by paired Student's t-test for the measures that were normally distributed, whereas the Wilcoxon test was used for measures that were not normally distributed, both with a significance level set at 5%.

## RESULTS

In order to test the hypothesis that the variables follow a normal distribution, the normality test of Kolmogorov-Smirnov, with a significance level set at 5%, was employed. It showed that all linear measurements presented normal distribution, which allowed the use of a parametric test (Student's t-test) when comparing the means. However, total facial convexity, nasofacial angles measurements and lip-chin proportion did not present normal distribution. For this reason, the non-parametric test of Mann-Whitney, with a significance level set at 5%, was used.

Random errors were detected in only two variables: angle of the lower third of the face and nasolabial angle. In assessing the systematic error, significant differences were found in only two out of 19 variables: angle of the lower third of the face and lip-chin proportion. As shown in Table 1, which expresses the statistical results of comparison between the linear measures of the two groups, all variables that assessed the position of the lower lip in relation to the reference lines (LL-S, LL H, LL-E, LL-B) and the distance Pn-H values, showed statistical significance differences (p < 0.05).

**Table 1:** Statistical results of comparison between groups.

Variables	Groups	n	Mean ± SD	P value
UL-S	Pleasant profile	30	-1.86 ± 1.34	0.176
	Unpleasant profile	30	-1.18 ± 2.35	
LL-S	Pleasant profile	30	-0.56 ± 1.50	0.014*
	Unpleasant profile	30	0.97 ± 2.93	
LL-H	Pleasant profile	30	0.41 ± 1.00	0.001*
	Unpleasant profile	30	1.77 ± 1.95	
Pn-H	Pleasant profile	30	9.52 ± 2.21	0.006*
	Unpleasant profile	30	11.80 ± 3.76	
LL-E	Pleasant profile	30	-2.02 ± 1.60	0.023*
	Unpleasant profile	30	-0.49 ± 3.18	
UL-E	Pleasant profile	30	-4.04 ± 1.60	0.259
	Unpleasant profile	30	-3.32 ± 3.07	
UL-B	Pleasant profile	30	3.59 ± 1.36	0.256
	Unpleasant profile	30	4.16 ± 2.39	
LL-B	Pleasant profile	30	2.75 ± 1.40	0.010*
	Unpleasant profile	30	4.36 ± 2.94	
Nasal length	Pleasant profile	30	2.45 ± 0.20	0.147
	Unpleasant profile	30	2.54 ± 0.28	
Tot. facial convex angle	Pleasant profile	30	142.67 ± 4.72	0.004^#^
	Unpleasant profile	30	139.10 ± 4.95	
Facial convex angle	Pleasant profile	30	169.20 ± 3.88	0.003*
	Unpleasant profile	30	165.17 ± 5.81	
Angle of the lower third	Pleasant profile	30	104.10 ± 6.63	0.006*
	Unpleasant profile	30	110.17 ± 9.49	
Nasofacial angle	Pleasant profile	30	32.73 ± 2.77	0.123
	Unpleasant profile	30	33.43 ± 3.01	
Nasolabial angle	Pleasant profile	30	104.37 ± 7.25	0.951
	Unpleasant profile	30	104.53 ± 12.91	
Mentolabial angle	Pleasant profile	30	137.10 ± 8.79	0.187
	Unpleasant profile	30	140.40 ± 10.31	
Total vertical proportion	Pleasant profile	30	0.95 ± 0.08	0.022*
	Unpleasant profile	30	1.01 ± 0.10	
Inferior third position	Pleasant profile	30	0.48 ± 0.05	0.600
	Unpleasant profile	30	0.47 ± 0.06	
Nasal height/length prop.	Pleasant profile	30	0.74 ± 0.09	0.400
	Unpleasant profile	30	0.72 ± 0.08	
Lip/chin prop.	Pleasant profile	30	1.34 ± 0.18	0.003^#^
	Unpleasant profile	30	1.58 ± 0.33	

* Statistical significance (p < 0.05) using Student's parametric t test;

# Statistical significance (p < 0.05) using Mann-Whitney non-parametric test.

The results presented in Table 1 show that the angular measurements (total facial convexity angle, facial convexity angle and angle of the lower third of the face), and the proportionality measurements (total vertical proportion and lip-chin proportion) showed p values < 0.05.

As for the assessment of sexual dimorphism, according to the Komogorov-Smirnov normality test at 5%, the following measures did not present a normal distribution in the pleasant profile group: angle of the lower third of the face, nasal length/ height and lip-chin proportions. In the unpleasant profile group, nasofacial, nasolabial and mentolabial angles did not have a normal distribution either.

Table 2 reveals sexual dimorphism in nasal length, nasofacial and angle of the lower third of the face, total vertical, nasal height/length proportions in the pleasant profile group. Differences between sexes were observed in the unpleasant profile group, in the linear measure nasal length, angle of the lower third of the face and the total vertical proportion.

**Table 2:** Statistical results of comparison between males and females of the pleasant and unpleasant profile groups.

Variables	Sex	Pleasant		Unpleasant	
		P value	p-valor	Mean ± SD	P value
UL-S	Male	-1.72 ± 1.26	0.587	-1.07 ± 1.74	0.815
	Female	-1.99 ± 1.45		-1.28 ± 2.90	
LL-S	Male	-0.87 ± 1.33	0.276	0.75 ± 2.65	0.684
	Female	-0.26 ± 1.64		1.19 ± 3.26	
LL-H	Male	0.10 ± 0.75	0.089	1.45 ± 2.04	0.378
	Female	0.72 ± 1.14		2.09 ± 1.88	
Pn-H	Male	9.75 ± 2.14	0.566	12.37 ± 3.58	0.411
	Female	9.28 ± 2.32		11.22 ± 3.98	
LL-E	Male	-2.35 ± 1.28	0.272	-0.75 ± 2.94	0.654
	Female	-1.69 ± 1.86		-0.22 ± 3.48	
UL-E	Male	-3.78 ± 1.57	0.382	-3.61 ± 2.15	0.606
	Female	-4.30 ± 1.64		-3.02 ± 3.84	
UL-B	Male	3.86 ± 1.27	0.278	4.27 ± 1.69	0.817
	Female	3.31 ± 1.44		4.06 ± 2.99	
LL-B	Male	2.55 ± 1.32	0.453	4.24 ± 2.66	0.823
	Female	2.95 ± 1.50		4.49 ± 3.29	
Nasal length	Male	2.57 ± 0.16	0.000*	2.69 ± 0.27	0.002*
	Female	2.33 ± 0.17		2.39 ± 0.20	
Tot. facial convex angle	Male	141.60 ± 5.30	0.222	139.53 ± 5.33	0.640
	Female	143.73 ± 3.95		138.67 ± 4.69	
Facial convex angle	Male	168.80 ± 4.44	0.581	165.40 ± 6.56	0.830
	Female	169.60 ± 3.33		164.93 ± 5.19	
Angle of the lower third	Male	108.00 ± 5.92	0.001*	115.20 ± 7.76	0.002*
	Female	100.20 ± 4.84		105.13 ± 8.48	
Nasofacial angle	Male	34.33 ± 2.66	0.001*	33.47 ± 3.72	0.629
	Female	31.13 ± 1.81		33.40 ± 2.23	
Nasolabial angle	Male	103.47 ± 7.02	0.506	105.13 ± 12.70	0.604
	Female	105.27 ± 7.61		103.93 ± 13.54	
Mentolabial angle	Male	137.47 ± 8.68	0.824	138.53 ± 10.33	0.290
	Female	136.73 ± 9.18		142.27 ± 10.29	
Total vertical proportion	Male	0.92 ± 0.09	0.032*	0.96 ± 0.08	0.019*
	Female	0.98 ± 0.05		1.05 ± 0.11	
Inferior third position	Male	0.48 ± 0.05	1.000	0.48 ± 0.05	0.247
	Female	0.48 ± 0.05		0.46 ± 0.06	
Nasal height/length prop.	Male	0.78 ± 0.10	0.001#	0.72 ± 0.07	0.636
	Female	0.69 ± 0.05		0.71 ± 0.10	
Lip/chin prop.	Male	1.35 ± 0.19	0.633	1.68 ± 0.38	0.073
	Female	1.34 ± 0.18		1.47 ± 0.22	

* Statistical significance (p < 0.05) using Student's parametric t test;

# Statistical significance (p < 0.05) using Mann-Whitney non-parametric test.

## DISCUSSION

Among the many goals of orthodontic treatment, proper teeth intercuspation and relationship with the skeletal bases, stability of results, correct occlusal function and, at present, due to the great emphasis that has been given to esthetics, a harmonious facial profile, have taken a prominent position on the objectives to be achieved at the end of orthodontic treatment.

In order to meet the esthetic expectations of patients, orthodontic treatment must include a detailed analysis of the facial profile. For many years, lateral cephalometric radiographs were used for this purpose. Standardized photographs have currently gained significant importance both clinically and in research, mainly because they reproduce the soft tissues in detail.^7,8,10,13,19^

When examining the error of the method, significant differences were found in three out of the 19 variables analyzed, which can be interpreted as having used a very reliable method. The measures angle of the lower third of the face, nasolabial angle and lip-chin proportion showed significant errors. Thus, due the difficulty of reproducibility and/or precision of these variables, they should be clinically interpreted with caution.

In other articles, references have been made to the difficulty of measuring the angle of the lower third (Sn-Me 'and Me'-C) and lip-chin proportion (Sn-Gn' and Gn'-C) due to the fact that both of them have the cervical point as reference, a point that is considered difficult to locate.^10,19^ The accumulation of adipose tissue in the neck is cited as a factor that hinders measurement of the angle of the lower third of the face^13^ Reche et al^19^ also point out the difficulty of measuring the nasolabial angle.

In this study, all linear measurements for the positioning of the lower lip in relation to the reference lines were statistically different between the pleasant and unpleasant groups. With regard to the distance of the lower lip to the S line, the pleasant group presented a profile with slight lip retrusion (mean -0.57 mm), which agrees with the findings of Carvalho^20^. Consequently, this study disagrees with the findings of Almeida et al^12^ who claim that, in both sexes and regardless of race, faces with lips touching the S line or with a slight protrusion are considered as pleasant. In recent research, Nomura et al^15^ investigated the preferences of evaluators of different ethnic groups assessing the positioning of the lips, and concluded that the African group of evaluators selected as pleasant those profiles with an average of -2.13 mm for the E line, a value that is very close to -2,02 mm obtained in this study.

The distance Pn-H also differs in the comparison between groups, and clinically expresses the amount of the nose that affects the profile. In other words, it represents the relationship between the nose, the upper lip and the chin, all of which are three important structures considered in the definition of a profile. It is worth noting that nasal length showed no significant difference between the profiles considered pleasant and unpleasant. For this reason, it can be deduced that the most important is not the size of the nose itself, but its relationship with other facial structures.

In the present study, the measures that assessed the influence of facial convexity over pleasantness presented significant differences between the pleasant and unpleasant groups. This difference was observed in both total facial convexity angle and angle of facial convexity and is in disagreement with Trevisan and Gil^8^ who found no significant differences in these variables between groups. However, these authors assessed patients with normal occlusion, whereas in this study, patient occlusion was not used as a criterion for inclusion or exclusion.

Data from this study corroborate the findings of a recent publication^21^ which found a strong association between facial profile esthetics and facial convexity angles, and deduced that profiles of which angle is increased or reduced are considered less esthetically pleasant. The total facial convexity angle assesses how the nose contributes to face convexity.^7^ In this study, the pleasant profiles mean of 142.37º (or 37.33º if we consider that some authors use the total value subtracted by 180º) agreed with the average of 38.93º found by Reche et al.^19^ The facial convexity angle determines the harmony of the face in the middle and lower thirds.^19^ In this study, the mean value for this angle was 169.2º (or 10.8º) in the pleasant group, a value that is very close to the average of 12º found among pleasant profiles investigated by Almeida et al.^12^

The angle of the lower third of the face assesses the protrusion of the chin in relation to the middle third of the face.^21^ It is of great importance in facial esthetics, and it appears, in another research^7^ as the third reason given by evaluators justifying an unpleasant profile. There is an agreement between the mean values obtained in this study and other similar studies,^13,21^ however, Trevisan and Gil^8^ found no significant differences for this variable between pleasant and unpleasant groups.

The total vertical proportion allows comparison between the height of the middle and lower thirds of the face, being the harmony and balance of the facial profile associated with a similar length of thirds.^13^ In our sample, the mean value for the pleasant profiles was 0.95, very close to that obtained in other studies.^13,21^ With regard to the lip-chin proportion, similar findings have been reported in the literature,^8^ but with male patients, only.

In assessing sexual dimorphism, differences were found for the length of the nose, nasofacial angle, angle of the lower third of the face and the total vertical and nasal height/length proportions. Sex differences in the linear measure of nasal length are expected, since males are, in a homogeneous way, larger than females in nearly every physical aspect.^22^ In a previous study, Reis et al^13^ observed differences for the variables angle of lower third face and total vertical proportion. Other authors,^9,23^ as well as in our study, did not find dimorphism regarding the position of the lips in relation to the reference lines.

In addition to the factors evaluated in this study, several others have an important influence over facial attractiveness and have already been referred to in the literature, namely: color of teeth, hair,^24^ lips, skin,^7,24^ nose, chin, jaws and eyes.^7^

## CONCLUSION

The anteroposterior position of the lower lip, the amount of the nose that affects the profile, the facial convexity and total vertical and lip-chin proportions seem to influence the pleasantness of facial profile. Sexual dimorphism was identified in the linear measure of nasal length, nasofacial angle, angle of the lower third of the face and the total vertical and nasal length/height proportions.
